# Burkitt Lymphoma Presenting With Acute Kidney Injury and Facial Nerve Palsy: A Case Report With Literature Review

**DOI:** 10.1002/ccr3.71363

**Published:** 2025-10-24

**Authors:** Gana Odeh, Fawwaz Yassin, Jalal Abuomar, Abdoh Abdallah

**Affiliations:** ^1^ An‐Najah National University Hospital Nablus Palestine; ^2^ Department of Pediatrics An‐Najah National University Hospital Nablus Palestine

**Keywords:** acute kidney injury, Burkitt leukemia, Burkitt lymphoma, facial nerve palsy, non‐Hodgkin lymphoma, pediatric oncology

## Abstract

Burkitt lymphoma is an aggressive non‐Hodgkin lymphoma. While kidney infiltration is well documented, acute kidney injury is an unusual presentation. Facial nerve palsy is even rarer. Here, we report a case of a 2‐year‐old boy with both of these unusual findings. He presented with acute kidney injury and bilateral nephromegaly. Two days later, he developed left‐sided facial nerve palsy. Imaging showed enlarged cervical lymph nodes. Bone marrow biopsy confirmed Burkitt lymphoma. He was treated following the COG ANHL01P1 protocol, with initial significant clinical improvement. Despite this, his condition deteriorated due to septic shock, and the patient died. This case emphasizes that Burkitt lymphoma can present with unusual symptoms. Therefore, it is important that clinicians maintain a high index of suspicion in cases with unusual constellations of symptoms, such as acute kidney injury and facial nerve palsy.

**Summary**

- Burkitt lymphoma is a non‐Hodgkin lymphoma that typically involves lymph nodes and the abdomen.
- Rare presentations, such as acute kidney injury and facial nerve palsy, may occur, highlighting the importance of a high index of suspicion and a multidisciplinary approach.

**Abbreviations**
BFM90: Berlin–Frankfurt–Münster 1990 protocol
BL: Burkitt lymphoma
BUN: blood urea nitrogen
CBC: complete blood count
COG ANHL01P1: Children's Oncology Group protocol ANHL01P1
CRP: C‐reactive protein
CSF: cerebrospinal fluid
EBV: Epstein–Barr Virus
EPOCH‐R: etoposide, prednisone, vincristine (oncovin), cyclophosphamide, doxorubicin (hydroxydaunorubicin), and rituximab
FABLMB 96: French–American–British/Lymphoma Malignancy B 1996 protocol
HIV: human immunodeficiency virus
IV: intravenous
LDH: lactate dehydrogenase
MRI: magnetic resonance imaging
NHL: Non‐Hodgkin lymphoma
VRE: vancomycin‐resistant *Enterococcus*

## 1 Introduction

Non‐Hodgkin lymphomas (NHL) are the most common lymphomas among children, accounting for around 60% of lymphoma cases [1]. Among the high‐grade NHL subtypes is Burkitt lymphoma (BL) [2]. It is an aggressive neoplasm, composed of B lymphocytes [3]. Its pathogenesis involves a chromosomal translocation of the c‐*myc* gene, most commonly *t*(8;14) translocation [4].

There are three identified clinical variants of BL: endemic, sporadic, and immunodeficiency‐associated. While the endemic variant often involves the head and neck, the sporadic form is more common for involving the abdomen [5].

BL has various clinical and radiological presentations [5]. It commonly involves lymph nodes and the abdomen, but bone marrow involvement is less common. This is a highly aggressive presentation, and when ≥ 25% neoplastic B‐cells are detected in the bone marrow, it is classified as Burkitt leukemia [6].

Even though bone marrow and CNS involvement are both associated with advanced stages, the prognosis of each differs [7]. Bone marrow involvement prognosis remains good, while CNS infiltration indicates a worse prognosis—which may be improved with rituximab therapy [6, 7, 8, 9]. CNS involvement in NHL is defined by the following criteria: any CNS tumor mass identified by imaging, cranial nerve palsy unexplained by an extradural lesion, or blasts detected in the cerebrospinal fluid (CSF) [7].

Furthermore, similar to other lymphomas, BL can result in kidney failure through different mechanisms. However, although the kidney is a common extrareticular and extrahematopoietic site of infiltration, acute kidney injury as an initial presentation of lymphomas remains rare [10].

Here, we report a case of a 2‐year‐old male child with BL, presenting with acute renal failure and unilateral facial palsy.

## 2 Case History/Examination

A 2‐year and 9‐month‐old male patient, with no significant past medical or surgical history, was referred from another hospital to the pediatric department at our hospital as a case of acute renal failure for nephrological evaluation and management. The family initially sought help at the referring hospital due to a 1‐day history of gait imbalance. He had a history of head trauma 1 week prior to admission, as well as an undocumented fever associated with tonsillitis, for which he had received antibiotics.

Due to the neurological symptoms and prior head trauma, a non‐contrast brain CT was performed at the referring hospital, which reported a basal skull fracture with maxillary sinus expansion. However, review at our hospital confirmed that there was no basal skull fracture, while verifying increased maxillary thickness and complete opacification of the middle ear cleft and mastoid air cells by fluid density. Incidentally, laboratory results at the referring hospital showed elevated kidney function tests, blood urea nitrogen (BUN) 48 mg/dL and creatinine 2.95 mg/dL.

On admission, vital signs were as follows: SpO_2_ 96%, blood pressure 130/98 mmHg, pulse 161 bpm, respiratory rate 21 breaths/min, and temperature 36.2°C.

On examination, the patient was stable, active, afebrile, and had unremarkable respiratory, neurological, cardiovascular, and gastrointestinal exams. However, signs of fluid overload were noted, including periorbital puffiness and bilateral lower limb pitting edema +2 according to the edema scale.

### 2.1 Investigations and Treatment

**FIGURE 1:**
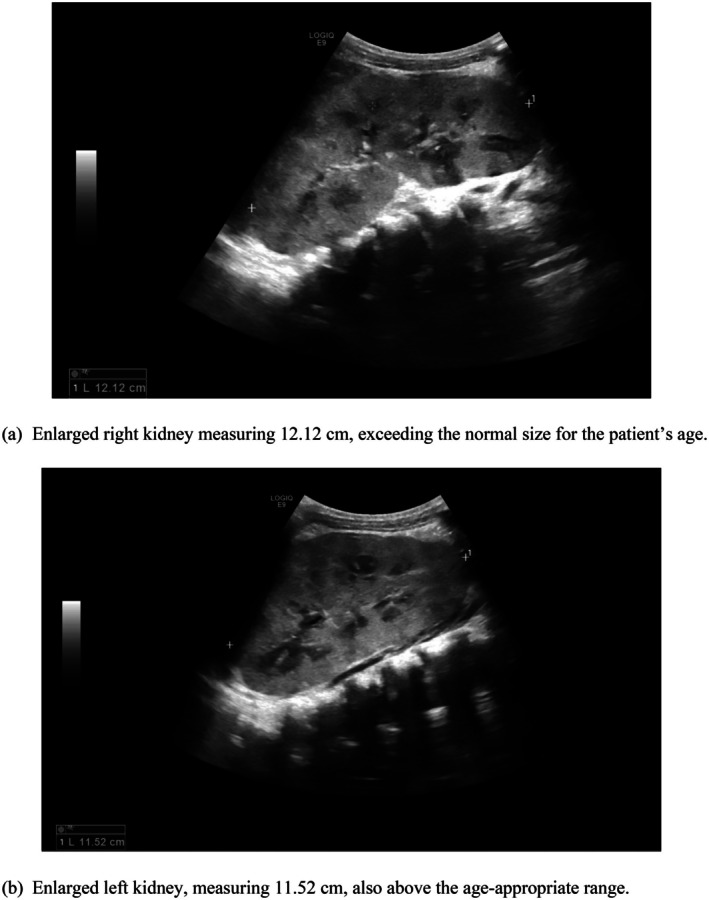
(a, b) Ultrasound of the kidneys upon admission. (a) Enlarged right kidney measuring 12.12 cm, exceeding the normal size for the patient's age. (b) Enlarged left kidney, measuring 11.52 cm, also above the age‐appropriate range.

The patient was admitted to the ward for nephrological evaluation and management. Abdominal ultrasound revealed bilaterally enlarged echogenic kidneys, with the right kidney measuring 12 cm and the left 11.5 cm in bipolar length, which are dramatically enlarged for his age (Figure 1). Laboratory tests showed the following: uric acid 10.5 mg/dL, BUN 51 mg/dL, creatinine 2.83 mg/dL, lactate dehydrogenase (LDH) 580 U/L, and C‐reactive protein (CRP) 60 mg/L. Complete blood count (CBC) was within normal limits. Arterial blood gases showed pH 7.45, PCO^2^ 19 mmHg, HCO^3^ 16.7 mmol/L, and base excess −11. Urinalysis revealed numerous white blood cells. A urine culture was obtained, and intravenous (IV) antibiotic and supportive therapy were initiated. Oral fluid intake was restricted to less than 500 mL per 24 h. A Foley catheter was inserted, and intake‐output monitoring was initiated.

**FIGURE 2:**
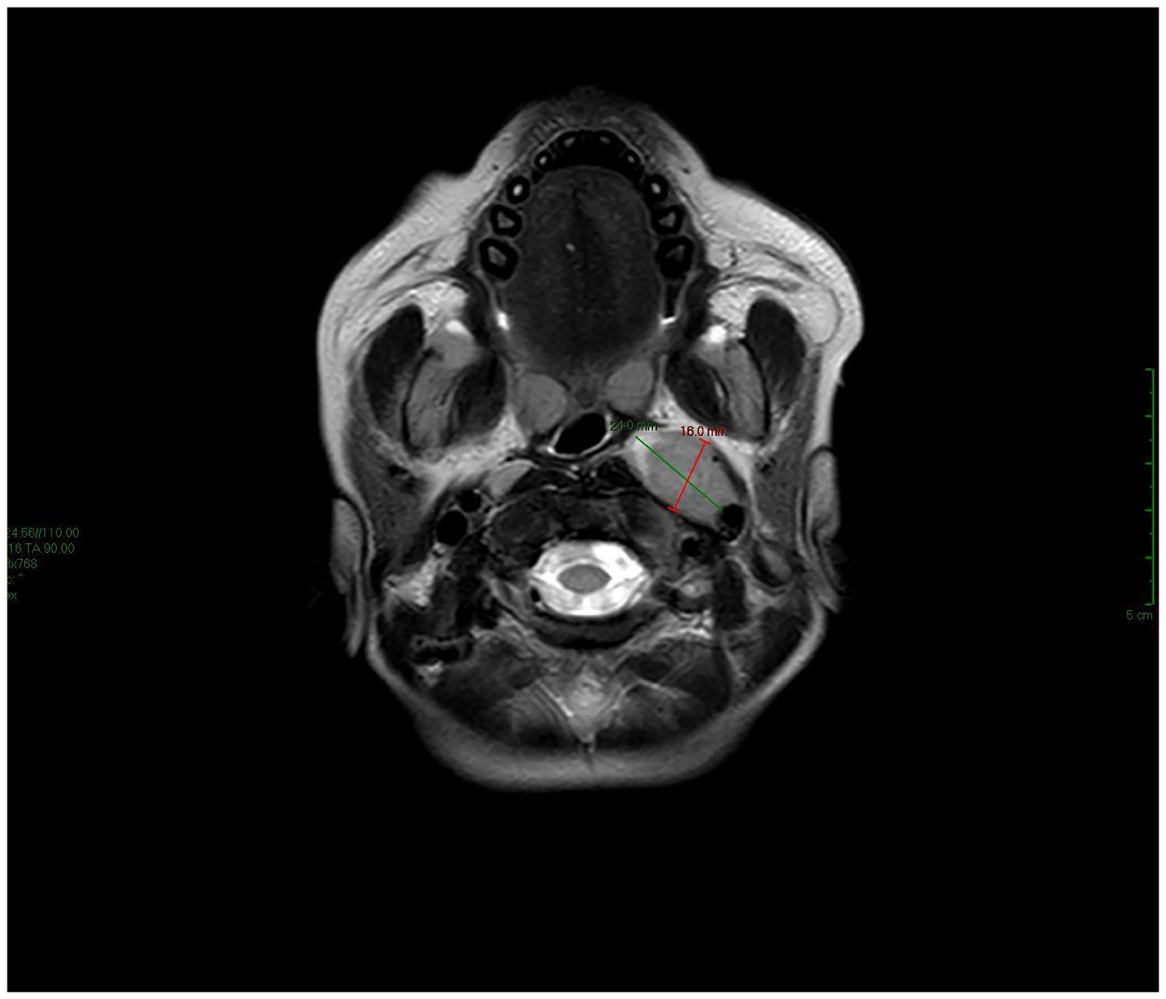
Brain MRI. Prominent cervical lymphadenopathy was noted, with the largest node measuring 24 × 16 mm.

**FIGURE 3:**
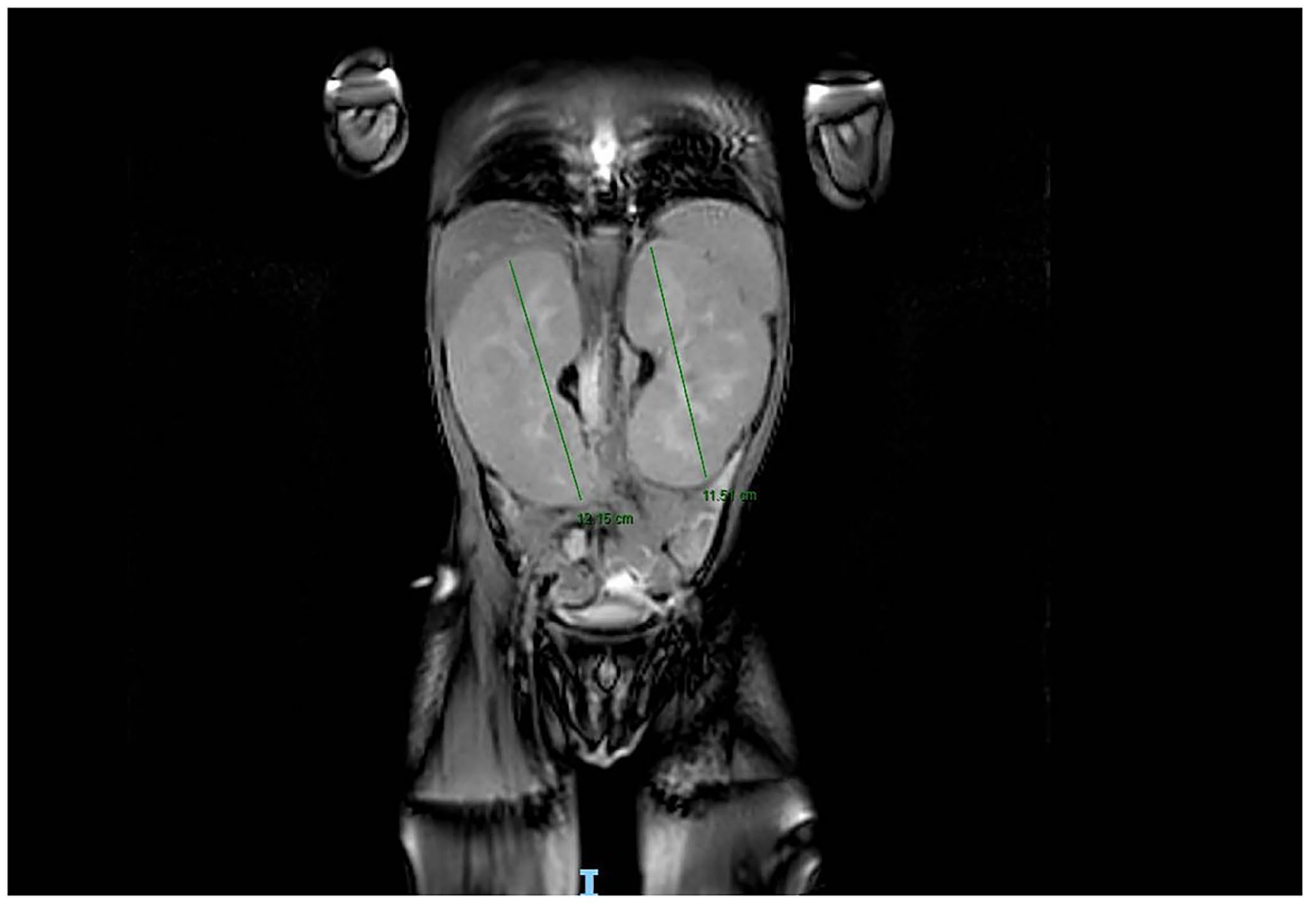
Abdominal MRI. Bilateral nephromegaly noted, with the right kidney measuring 12.15 cm and the left 11.51 cm.

Two days later, the patient developed left‐sided facial nerve palsy, graded as House‐Brackmann Grade IV. Neurological examination was otherwise normal. Brain and abdominal magnetic resonance imaging (MRIs) were ordered. The brain MRI showed prominent bilateral cervical lymph nodes, with the largest being a left parapharyngeal node measuring approximately 2.4 × 1.6 cm (Figure 2). There was also diffuse, bilateral, and symmetrical soft tissue thickening in the maxillary sinuses, suggestive of extramedullary hematopoiesis, consistent with the previous CT findings. Otherwise, the brain MRI was unremarkable. The abdominal MRI revealed marked diffuse enlargement of both kidneys: the right measured 13 cm in bipolar length and the left 12.5 cm, both abnormal for the patient's age (Figure 3). The liver and spleen were normal. Findings were suggestive of BL.

Kidney biopsy was performed without immediate complications. Histopathology revealed involvement by high‐grade lymphoma, confirmed to be BL. Additionally, the patient underwent an urgent bone marrow biopsy and lumbar puncture. The CSF cytology was negative for morphologically atypical cells.

Follow‐up labs showed worsening renal function: BUN 75.5 mg/dL and creatinine 2.4 mg/dL. The patient was oliguric. In light of elevated LDH, worsening renal function, hyperuricemia, and metabolic disturbances, tumor lysis syndrome was suspected and management with allopurinol and hydration was initiated accordingly.

Following these procedures, IV methylprednisolone 10 mg was initiated. A temporary dialysis catheter was inserted the same day, and hemodialysis was started.

The peripheral blood smear showed mildly decreased red blood cell count with normocytic, normochromic features and rare nucleated red blood cells. No schistocytes were seen. White blood cells were elevated, with occasional immature myeloid cells noted. No definite blasts were identified. Platelets were adequate, with no aggregates seen. Impression: Leukoerythroblastic blood film.

Bone marrow biopsy revealed extensive infiltration by high‐grade B‐cell lymphoma. The immunophenotype was consistent with BL. Flow cytometry and immunohistochemistry studies were performed. Flow cytometry demonstrated lymphoid cells expressing CD20 and CD10. Immunohistochemistry showed that the lymphoid cells were positive for CD20, CD10, BCL6, and c‐MYC (expressed in ~80% of cells), and negative for BCL2, MUM1, CD34, and TdT. The Ki‐67 proliferation index was elevated at approximately 95%–100%. CD3 stains a few background T cells. The patient was diagnosed with Burkitt leukemia and started chemotherapy treatment according to the Children's Oncology Group—A Non‐Hodgkin Lymphoma (COG ANHL01P1) Protocol. He had elevated blood pressure (above the 95th percentile +12), which was controlled with oral amlodipine and oral propranolol. He required several red blood cell and platelet transfusions. His kidney function tests improved during management. Last renal ultrasound showed improvement in kidney size, yet they are still large for his age. His facial palsy improved from Grade IV to Grade I.

### 2.2 Outcome and Follow‐Up

Overall, since the diagnosis, treatment has proceeded well with no delays. This was assessed regularly through follow‐up visits and laboratory testing of renal function and blood counts. After each chemotherapy cycle, he was assessed and monitored closely for potential side effects and managed promptly. Complications such as tumor lysis syndrome, neutropenic fever, and mucositis occurred and were managed effectively.

Although he initially showed significant clinical improvement, during the third month after first admission, his condition deteriorated. Following a chemotherapy session, the patient developed Vancomycin‐resistant *Enterococcus* (VRE)–induced decompensated septic shock and died.

The timeline of major case events is summarized in Table 1.

**TABLE 1:** Chronological summary of case events.

Timeline	Clinical events
Day 1	Referred as a case of acute kidney injury for nephrological evaluation. Signs of fluid overload were noted Labs upon admission showed AKI; imaging revealed bilateral nephromegaly
Day 3	Left‐sided facial nerve palsy Grade IV
Day 4	Brain and abdominal MRIs were ordered, showing prominent bilateral cervical lymph nodes, and diffuse enlargement of both kidneys, respectively
Day 5	Bone marrow and kidney biopsies were performed
Days 5 + 6 + 7	Hemodialysis sessions were performed
Day 7	Biopsy confirmed Burkitt lymphoma with marrow involvement
Week 2	Initiated treatment: COG ANHL01P1 protocol
Month 1	Continued chemotherapy and intrathecal therapy
Month 2	Stable renal function, mild residual facial asymmetry
Month 3	Sudden deterioration and death due to VRE‐induced septic shock

## 3 Discussion

BL, an aggressive non‐Hodgkin lymphoma, accounts for approximately 40% of pediatric NHL cases in the United States and 30%–50% of all pediatric lymphoma cases [11, 12]. NHLs in children typically present with involvement of the abdomen, mediastinum, and/or head and neck, in addition to involvement of the bone marrow or CNS [11].

BL is classified into three clinical variants: endemic, sporadic, or immunodeficiency‐related. The endemic form mostly occurs in equatorial Africa and is associated with Epstein–Barr virus (EBV) infection and head and neck involvement. The sporadic variant is more common in non‐endemic regions and more commonly presents with abdominal involvement; it can also be associated with EBV infection. The immunodeficiency‐associated form occurs in patients with conditions leading to immune suppression, such as human immunodeficiency virus (HIV) infection, organ transplants, or congenital immunodeficiency syndromes [5, 13, 14].

The case reported herein describes a rare and diagnostically challenging presentation: a 2‐year‐old child with BL who presented with acute renal failure and facial nerve palsy—a highly atypical combination of symptoms.

### 3.1 Renal Involvement and Acute Kidney Injury

AKI can occur in cancer patients through different mechanisms, including volume depletion, urinary tract obstruction, infiltration of the kidneys, and tumor lysis syndrome [15]. AKI secondary to infiltration by hematological malignancies is rare. It is estimated that only 1% of acute leukemia cases present in this manner, while chronic leukemias and lymphomas are even less commonly associated with this presentation [10].

In our case, the patient's presentation of bilateral nephromegaly suggested infiltration as the etiology of AKI, which was later confirmed by kidney biopsy. Improvement in kidney function and size after temporary dialysis and chemotherapy sessions further supported the diagnosis.

A review of the literature identified cases in which AKI was the initial presentation of BL. For example, Ter Haar et al. [16] reported a 4‐year‐old male case who presented with bilateral nephromegaly, malignant hypertension, and AKI; renal infiltration was confirmed through kidney biopsy. Similarly, Caballero et al. [17] discussed a 6‐year‐old male with bilateral renal masses and biopsy‐confirmed BL. In another pediatric case with a worse outcome, an 8‐year‐old girl presented with acute renal failure; BL was confirmed by renal biopsy, but the patient died before receiving chemotherapy [18].

Furthermore, Ninh et al. [19] documented a case with bilateral nephromegaly, hypertension, and AKI without systemic lymphadenopathy, and it was diagnosed as primary renal BL. Consistent with the latter case, Agarwal et al. [20] reported a 4‐year‐old boy with bilateral renal masses and abnormal kidney function, diagnosed as primary Burkitt kidney lymphoma.

These cases overlap in their presentation with our reported case but differ in the underlying mechanism. While three of them, along with our case, had AKI resulting from secondary BL infiltration [16, 17, 18], AKI in the other two cases resulted from primary renal BL [19, 20]. Details of the cases in comparison are summarized in Table 2.

**TABLE 2:** Literature review of BL cases presenting with AKI.

Case	Year	Age (years)	Presentation	Diagnosis	Treatment	Outcome	References
1	2016	Four	Renal: Bilateral nephromegaly, hypertension, severe AKI Others: General weakness, anorexia, vomiting and abdominal pain	Renal biopsy confirmed Burkitt lymphoma	Chemotherapy: according to the Inter‐B‐NHL rituxan 2010 protocol Hemodialysis: was required due to tumor lysis syndrome	Renal function improved	[16]
2	2023	Six	Renal: diffuse lump in the left flank Others: Abdominal pain, nausea with vomiting, anorexia, weight loss, and night fever	Renal biopsy confirmed Burkitt lymphoma	ANHL 1131 protocol	Complete remission after 2nd induction	[17]
3	2022	Eight	Renal: AKI, edema Others: anterior cervical swelling and fever	Renal biopsy confirmed Burkitt lymphoma	Required several blood transfusions and hemodialysis sessions; however, the patient died before chemotherapy could be initiated	Fatal outcome	[18]
4	2021	Four	Renal: oliguria, bilateral nephromegaly Others: Abdominal pain, vomiting, weakness, and anorexia	Renal biopsy confirmed Burkitt lymphoma	EPOCH‐R chemotherapy, normalization of renal size	Both kidneys returned to normal size	[19]
5	2015	Four	Renal: AKI, bilateral lower abdominal lump Others: abdominal distension, abdominal pain, and fever	Renal biopsy confirmed Burkitt lymphoma	French‐American‐British/lymphoma malignancy B (FAB‐LMB) 96 treatment protocol	Renal function improved	[20]
6	2025	Two	Renal: AKI, bilateral nephromegaly, periorbital puffiness, and bilateral lower limb pitting edema Others: Left‐sided facial nerve palsy	Renal biopsy confirmed Burkitt lymphoma	Chemotherapy: COG ANHL01P1 chemotherapy protocol Hemodialysis	Initial improvement, followed by deterioration and death	Present case

Even though the case presented with AKI and nephromegaly primarily, cervical lymphadenopathy was identified through imaging, and bone marrow involvement was detected. Consequently, the case does not meet the criteria for primary renal BL as defined by Malbrain and Stallone [21, 22].

Together, all these cases emphasize the importance of considering BL in pediatric patients as the culprit in unexplained AKI and bilateral nephromegaly, even in the absence of systemic findings.

In addition to the renal findings, our case also presented with facial nerve palsy, a much rarer manifestation of BL, which is addressed in the following section.

### 3.2 Facial Nerve Palsy: A Rare Neurologic Presentation

CNS involvement occurs in 5%–40% of patients with BL [23]. Clinical presentations vary and may include cranial nerve palsy, plegia, seizures, altered consciousness, and other neurologic signs [24, 25]. The pathogenesis of cranial nerve and meningeal involvement is still not fully established. It is speculated that migratory extension to the dura may occur directly through bone and periosteum, or along nerve or blood vessel sheaths [24].

Facial nerve palsy as an initial presentation is exceedingly rare, and very few cases have been documented [26, 27]. A limited number of pediatric cases have described it as an early symptom of BL. A case series by Amoils et al. [27] mentioned two pediatric patients with the unusual presentation of facial paresis. However, both cases showed temporal bone involvement and middle ear findings, which were not stated in our case. Similarly, another series reported by Hong et al. [28] documented two cases with facial palsy associated with other rhinologic symptoms—not evident in our case. It is also worth mentioning the case reported by Nesheli of a 3‐year‐old boy with painful proptosis of the left eye and sixth and seventh cranial nerve palsies. This case differs from the others, as investigations confirmed it to be primary CNS BL [29]. Table 3 summarizes the comparative details of the cases.

**TABLE 3:** Literature review of BL cases presenting with facial nerve palsy.

Case	Year	Age (years)	Presentation	Diagnosis	Treatment	Outcome	References
1	2016	Three	Symptoms: Worsening headaches, right facial palsy, and progressive nasal congestion	Biopsy of the nasopharyngeal mass confirmed Burkitt lymphoma	ANHL 1131 chemotherapy protocol	Facial nerve function had recovered to Grade I/VI by the end of consolidation chemotherapy	[27]
2	2016	Two	Right facial paralysis: Initially misdiagnosed as Bell's palsy and otitis media; ultimately attributed to malignant infiltration	Biopsy of the mastoid and middle ear confirmed Burkitt lymphoma	ANHL 1131 chemotherapy protocol	Facial nerve function improved to a grade of II/VI by the completion of treatment	
3	2019	Four	Symptoms: Progressive right facial palsy, intermittent facial pain, periodic epistaxis, dysphonia	Biopsy of the middle ear mass confirmed the diagnosis of Burkitt lymphoma	A chemotherapy regimen consisting of methotrexate, cytarabine, and etoposide	Twelve years later, there was no clinical or radiologic recurrence, nor any complications	[28]
4	2019	Three	Symptoms: Left eye pain, right eye ptosis, left facial numbness on examination.	Biopsy through endoscopic sinus surgery confirmed Burkitt lymphoma	Clinical trial COG protocol ANHL 1131 chemotherapy protocol	In remission from the disease for 5 years	
5	2015	Three	Findings: Left neck mass, painful left eye proptosis. Neurologic signs: Cranial nerve VI and VII palsies.	Biopsy of paranasal sinus confirmed the diagnosis of Burkitt lymphoma	BFM‐90 chemotherapy protocol	Complete remission	[29]
6	2025	Two	Referred for nephrological management. Left‐sided facial nerve palsy 2 days after admission	Diagnosis was confirmed through bone marrow biopsy, flow cytometry, and kidney biopsy. No nasopharyngeal or sinus biopsies were performed	COG ANHL01P1 chemotherapy protocol	Initial improvement, followed by deterioration and death	Present case

In contrast to previously reported cases, our patient presented with facial nerve palsy despite no radiographic evidence of skull base or middle ear involvement. Neurologic symptoms like facial palsy can be deceptively straightforward. Clinicians often first consider common and self‐limiting causes such as Bell's palsy, viral infection, or otitis media. But our case reminds us that when facial nerve palsy persists or appears alongside other concerning signs—like acute kidney injury or lymphadenopathy—more serious pathology must be considered, and advanced imaging should not be delayed.

The literature, combined with our findings, suggests the importance of not isolating these symptoms from each other. It is essential to perform advanced imaging early in the course of the disease, even when signs are mild or nonspecific. This was our approach in the case, using renal ultrasound and performing both abdominal and brain MRIs—each of which proved essential. Not to forget the biopsy, which helped further confirm the suspected diagnosis. Later on, staging becomes an important step through lumbar puncture and bone marrow analysis to determine the extent of disease and tailor the best treatment plan.

Fortunately, once the diagnosis is made, there are established protocols that work. Regimens like the one used in our patient or in other mentioned cases, combined with intrathecal therapy when indicated, offer strong outcomes when treatment is started promptly. However, poor outcomes can arise during the course of management that might threaten life and lead to death, as in this case of death due to septic shock. Septic shock remains a major cause of death in pediatric cancer patients [30].

The study was limited by the absence of confirmed mass invasion or abnormal CSF analysis to confirm BL as the cause of facial nerve palsy. Nevertheless, the presence of facial nerve palsy in the absence of an extradural lesion may still support BL as an underlying etiology, given that this presentation is included in the CNS involvement criteria for NHLs.

## 4 Conclusion

Burkitt lymphoma can present with deceiving symptoms. Rare presentations as acute kidney injury and facial nerve palsy, especially when combined, should prompt further investigations. Rapid management with an appropriate chemotherapy protocol can lead to significant improvement, but adverse outcomes can still happen and should be anticipated. This case adds to the limited literature, showing the need for a low threshold of clinical suspicion and the importance of a multidisciplinary diagnostic approach involving both radiology and tissue biopsy.

## Author Contributions

**Gana Odeh:** conceptualization, data curation, project administration, writing – original draft, writing – review and editing. **Fawwaz Yassin:** conceptualization, investigation, supervision, writing – review and editing. **Jalal Abuomar:** writing – original draft, writing – review and editing. **Abdoh Abdallah:** data curation, investigation, project administration, supervision, writing – review and editing.

## Consent

Written informed consent was obtained from the patient's legal guardian, as the patient is a minor.

## Conflicts of Interest

The authors declare no conflicts of interest.

## Data Availability

The information used is not publicly available to ensure patient confidentiality. Relevant details can be provided by the corresponding author upon reasonable request, in accordance with privacy and ethical guidelines.
